# Small metabolites vary in sow milk across the course of lactation, while moringa supplementation and cooling sows exposed to heat stress conditions have limited effects

**DOI:** 10.1093/tas/txag007

**Published:** 2026-02-20

**Authors:** Leriana Garcia Reis, McKeeley C Stansberry, Wonders Ogundare, Evy Tobolski, Linda M Beckett, Allan P Schinckel, Christina Ramires Ferreira, Radiah C Minor, Theresa Casey

**Affiliations:** Department of Animal Sciences, Purdue University, West Lafayette, IN 47907, United States; Department of Animal Sciences, Purdue University, West Lafayette, IN 47907, United States; Department of Animal Sciences, Purdue University, West Lafayette, IN 47907, United States; Department of Animal Sciences, Purdue University, West Lafayette, IN 47907, United States; Department of Animal Sciences, Purdue University, West Lafayette, IN 47907, United States; Department of Animal Sciences, Purdue University, West Lafayette, IN 47907, United States; Bindley Bioscience Center, Purdue University, West Lafayette, IN 47907, United States; Department of Animal Sciences, North Carolina Agricultural and Technical State University, Greensboro, NC 27411, United States; Department of Animal Sciences, Purdue University, West Lafayette, IN 47907, United States

**Keywords:** colostrum, mammary gland physiology, metabolic pathways, milk metabolomics, neonatal nutrition

## Abstract

This study aimed to characterize changes in small metabolites in sow milk over the course of lactation. The impact of *Moringa oleifera* leaf powder addition to diet and electronic cooling pads (ECP) on milk metabolites of sow under moderate heat stress was also evaluated. A 2 × 2 factorial experiment was conducted with Yorkshire x Landrace sows (*n* = 48) from gestation day 100 to lactation day 21. Treatments included heat stress + control diet (HS + CS), heat stress + *Moringa* (HS + M), ECP + CS, and ECP + M beginning with 12 sows per treatment. Milk was collected on lactation days 0 (D0, colostrum), 3 (D3, transitional milk), and 14 (D14, mature milk). Metabolites were extracted using the Bligh and Dyer method and then profiled using exploratory multiple reaction monitoring. Milk metabolite content varied significantly across lactation days. Diet and parity influenced milk metabolites on D0, ECP on D3, and parity on D14. The 55 metabolites increased between D0 and D3, linked to phenylalanine, tyrosine and tryptophan biosynthesis, starch, sucrose and galactose metabolism, while the 93 decreased metabolites were associated with protein synthesis and gut development, including phenylalanine, tyrosine and tryptophan biosynthesis and alanine, aspartate and glutamate metabolism. Between D3 and D14, 148 metabolites increased and reflected alanine, aspartate and glutamate and galactose metabolism, while the 21 decreased included L-leucine, creatine, myo-inositol, hypoxanthine and acetyl-carnitine. The 116 metabolites impacted by parity in D0 samples, were elevated in primiparous compared to multiparous sows, and linked to arginine biosynthesis, amino acid metabolism, and purine metabolism. On D14, parity affected 52 metabolites related to alanine, aspartate and glutamate metabolism, arginine biosynthesis and pyrimidine metabolism. ECP on D3 reduced 104 metabolites involved in the citrate acid cycle, cysteine and methionine metabolism, and pyrimidine metabolism. Milk metabolite content changed significantly between the three phases of milk production, reflecting mammary secretory activity and potentially the changing nutritional needs of piglets and maternal physiological adjustments throughout lactation. These findings highlight the dynamic nature of milk composition and the potential for dietary and environmental interventions to modulate milk metabolite content under heat stress conditions.

## Teaser

This study shows how sow milk composition shifts throughout lactation and demonstrates that diet and cooling strategies can influence milk quality under heat stress, offering potential benefits for piglet growth and sow health.

## Graphical abstract

**Graphical Abstract:**
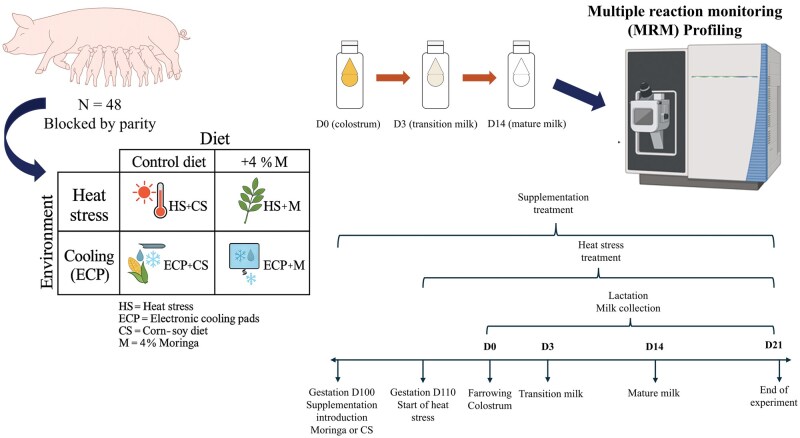

## Introduction

Milk is a complex biological fluid that changes in composition across the course of lactation reflecting the synthetic activity of the mammary gland as well as the nutritional needs of neonates. In addition to proteins, lipids, and lactose in milk, there are a multitude of small metabolites including amino acids, monosaccharides, nucleic acids and microbial-derived metabolites (Ojo‐okunola et al. 2020; Poulsen et al. 2022; Connolly et al. 2023). Similar to changes in protein, lactose, and lipid content of milk (Bradshaw et al. 2021; Suarez-Trujillo et al. 2021; Rajput et al. 2023), milk metabolite composition undergoes dynamic changes throughout the course of lactation. Studies of human and bovine milk indicate that changes in milk metabolite composition across the course of lactation reflect maternal metabolism and may affect neonate immune function, energy metabolism, and gut microbiota development (Poulsen et al. 2022; Connolly et al. 2023). A study of sow milk using ^1^H-NMR spectroscopy demonstrated that metabolite profiles vary depending on lactation stage and parity. The concentrations of creatine, creatine phosphate, glutamate, and glycolate were particularly affected by the interaction between days postpartum and sow parity (Settachaimongkon et al. 2023).

The phases of milk production in eutherian mammals, which include swine, are described as colostrum, transitional and mature milk. Colostrum contains higher concentrations of immunoglobulins, more protein, differential ion balance, higher concentrations of cytokines and growth factors, and low levels of lactose compared to mature milk, with transitional milk composition being distinct from and intermediate to colostrum and mature milk (Suarez-Trujillo et al. 2021; Bradshaw et al. 2021; Rajput et al. 2023). Differences in composition of milk between the phases of milk production reflect, at least in part, the differences in mammary gland development and activity of milk producing lactocytes.

Periparturient hormonal changes, including the drop in progesteronse and the rise in glucocorticoids and prolactin, initiate secretory activation in the mammary gland (Casey and Plaut 2007). During secretory activation, the lactocyte’s capacity for de novo fatty acid synthesis is initiated and lactose synthesis increases. Structural changes also occur in lactocytes, to include closure of tight junctions between epithelial cells that result in the formation of the blood-milk barrier. The blood–milk barrier prevents blood components from entering mammary secretions, resulting in lower concentrations of sodium, chloride, plasma proteins, antibodies, and leukocytes in the milk (Wellnitz and Bruckmaier 2021). Lactose functions as an osmolyte. Increasing lactose concentrations with secretory activation promotes water influx into the mammary secretion, resulting in overall dilution of the milk. Citrate concentrations also increase in milk as secretory activation increases. Citrate is a primary substrate for fatty acid synthesis, and so increasing concentrations reflect the gain in the lactocyte’s capacity for de novo fatty acid synthesis (Rudolph et al. 2007). The transition from colostrum to mature milk also involves maternal metabolic shifts, with increase in energy demand by mammary epithelial cells to support lactose, lipids, and protein synthesis (Casey et al. 2000; Lee and Kelleher 2016). Therefore, given the combined structural changes of the lactocytes and increasing nutrient demands by mammary epithelial cells to support milk component synthesis, the abundance of small metabolites in milk may change throughout lactation.

Milk composition is also shaped by genetic, physiological, and environmental factors, which collectively determine its nutritional quality and functional properties. Parity influences yield, with multiparous sows producing more milk. Genetics and breed affect fat and protein levels, and aging reduces fat and solids-not-fat content [Products, National Research Council (US) Committee on Technological Options to Improve the Nutritional Attributes of Animal 1988; Bondan et al. 2021; Bednarski and Kupczyński 2024]. Among extrinsic factors, diet alters fat content, while environmental conditions like temperature and humidity impact milk composition (Alothman et al. 2019). Poor health status can reduce milk quality, and management practices, and factors like suckling or milking frequency can affect quality and yield (Mylostyvyi and Chernenko 2019; Marumo et al. 2022; Toghdory et al. 2022).

Heat stress (HS) in swine leads to decreased feed consumption, poor nutrient digestion, reduced weight gain, leaky gut, increased susceptibility to infectious disease, and increased mortality (Ross et al. 2015). In addition to these negative health and production outcomes, gestating and lactating sows experiencing HS also produce less milk. Although the 60 to 70% depression in feed intake during HS is the primary reason for decreased milk production (Collier et al. 2017), other factors contribute to the depression in milk production, such as metabolic adaptations to HS across the whole body. These include changes in carbohydrate and lipid metabolism (Wheelock et al. 2010; Liu et al. 2017), which ultimately lead to decreased milk production. Heat stress also drives oxidant stress and leads to elevated levels of free radicals in tissues of HS animals, which causes both cellular and mitochondrial oxidative damage to mammary tissues. Combined, alterations in nutrient supply to the mammary gland and in mammary cell oxidative state may result in alterations in milk metabolites. *Moringa oleifera* and electronic cooling pads (ECP) are promising dietary and management interventions for mitigating heat stress, and its adverse effects on animal health and productivity. *Moringa*, commonly known as the “miracle tree,” is rich in antioxidants, vitamins, and bioactive compounds that combat oxidative stress, improve immune function, and enhance nutrient utilization (Chumark et al. 2008; Ayirezang et al. 2020; Pareek et al. 2023). *Moringa* leaf consumption during gestation and lactation was shown to positively impact milk quantity and quality in multiple species (Mendieta-Araica et al. 2011; Babiker et al. 2017; Zhang T. et al. 2018; Kholif et al. 2019; Zeng et al. 2019; Mutwedu et al. 2022). Our studies showed that dietary supplementation with *Moringa* impacted milk macronutrient composition and reduced oxidative damage in heat-stressed sows (Stansberry et al. 2024). Meanwhile, ECP offer a technological approach to heat stress alleviation through conductive cooling, effectively lowering respiration rates, rectal temperatures, and body weight loss in lactating sows while increasing feed intake (Ogundare et al. 2025). Notably, the combination of *Moringa* supplementation and ECP use has been found to synergistically enhance milk composition, particularly by increasing fat content (Stansberry et al. 2024; Reis et al. in review). These findings highlight the potential of integrating dietary antioxidants with cooling technologies to improve animal welfare and productivity, particularly in the face of rising global temperatures.

Herein, we apply exploratory multiple reaction monitoring (MRM) profiling to characterize changes in milk metabolite composition of sows across lactation stages (Edwards et al. 2021). We also analyzed the effects of *Moringa* supplementation, conductive cooling with ECP and parity on milk metabolites in sows exposed to moderate heat stress. MRM-profiling is a highly sensitive mass-spec scan of tentatively attributed molecules based on mass to charge ratios of precursor ions and ionization products (Edwards et al. 2021; Reis et al. 2023). By analyzing metabolic changes across different lactation stages, we sought to identify key temporal shifts in milk composition related to mammary gland activity and developmental needs of neonates. Analysis of the impact of *Moringa* supplementation and conductive cooling with ECP on milk metabolites of moderately heat stressed sows aimed to add to the understanding of how these factors alone and together affect milk composition and mammary gland function in heat-stressed environments.

## Material and methods

### Animals and experimental design

**Fig. 1.**
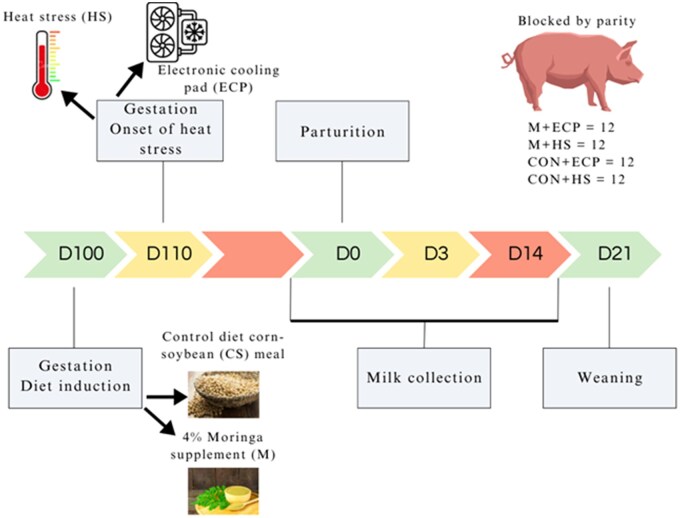
Experimental design of the 2 × 2 factorial study. The experimental design employed a 2 × 2 factorial structure with 48 mixed-parity yorkshire × landrace sows, mated to duroc sires. The sows were blocked by parity and randomly allocated to one of four treatment groups: a) heat stress with a corn-soybean meal control diet (HS + CS), b) heat stress with a moringa-supplemented diet (HS + M), c) cooling with ECP and the control diet (ECP + CS), and d) cooling with ECP and the moringa-supplemented diet (ECP + M). The female’s sows were housed collectively in a farrowing barn, and the experimental diets were introduced at ∼100 days of gestation. Heat stress and cooling interventions were applied from late gestation (day 110—D110) through to wmeaning (dmay 21—D21 of lactation). Conductive cooling was facilitated by placing ECP mats under the farrowing crates for the sows to lie on. To induce moderate heat stress, room temperatures were adjusted to mimic the conditions of a hot summer day. Temperatures were incrementally increased from 26°C to 32°C between 0800 and 1100, maintained at 32°C until 1700, then gradually decreased to 26°C by 2000, with this cycle repeated daily. Milk samples were manually collected from sows at lactation day 0 (D0), day 3 (D3), and day 14 (D14) to represent colostrum, transitional, and mature milk stages. The milk samples were aliquoted and stored at -20°C for subsequent analysis of metabolites using MRM-profiling.

All animal procedures and experimental design methods were reviewed and approved by the Purdue University Animal Care and Use Committee (Protocol Number: 2110002202) before the start of the study. This study was part of a larger experiment, detailed in previous publications (Stansberry et al. 2024; Ogundare et al. 2025; Tobolski et al. 2025). Briefly, a 2 × 2 factorial design was employed using 48 mixed-parity Yorkshire × Landrace sows mated to Duroc sires. The sows were randomly assigned to one of four treatment groups (*n* = 12 per treatment), with two replicates (*n* = 6 sows per treatment per replicate, Fig. 1). All sows within each replicate were housed in the same room. Moderate heat stress conditions to match temperature changes across a summer day were applied from late gestation (day 100) to weaning (day 21 of lactation). The treatment groups included: i) heat stress with a corn-soybean meal control diet (HS + CS), ii) heat stress with a corn-soybean meal diet supplemented with 4% ground *Moringa oleifera* leaf powder (HS + M), iii) cooling with ECP and the control diet (ECP + CS), and iv) cooling with ECP and the 4% *Moringa*-supplemented diet (ECP + M), with *n* = 6 primiparous sows in each treatment group. Multiparous sows averaged 5 ± 1.05 parities. The first replicate was conducted from August 15, 2022 to September 20, 2022, and the second from October 12, 2022 to November 15, 2022.

Diets were introduced at approximately 100 days of gestation, 9 to 10 days before heat stress exposure. The control diet consisted of a corn-soybean meal formulation, balanced to meet or exceed NRC requirements for gestation and lactation. To prepare the *Moringa* diets, 4% ground *Moringa* leaf powder (Jimfina Farm; Brown Summit, NC, USA) was added to the control diet, which was modified to ensure equivalent metabolizable energy (ME) content (Table 1). In particular, corn, soybean meal, and white grease were 58.8%, 34% and 3% of CS control diet, and 54.2%, 33.1% and 4.7% of the *Moringa* diet, respectively. The changes resulted in differences in percent fat of analyzed diet, with CS lactation diet being 5.5% fat and *Moringa* diet being 7.7%. The crude protein content of lactation diets remained similar between diets, with CS at 20.5% and *Moringa* at 21.5%. Details on the diet formulations and *Moringa* leaf compositional analysis are provided in our previous publication (Stansberry et al. 2024; Reis et al. in review), and further information regarding diet and *Moringa* leaf lipidome analysis can be found at DOI: doi : 10.4231/HVSB-P274 (Beckett et al. 2025). During gestation, sows were limit-fed 2.72 kg/day, divided into two meals at 0700 and 2000. The transition to lactation diets began on gestation days 109 to 111, and after farrowing, sows were allowed ad libitum access to feed. Litters were standardized to 13 piglets per sow within 24 hours of birth through cross-fostering within treatment groups.

**Table 1.** Gestation and lactation diet^a^ composition of control and moringa diets (as-fed basis). The table was adapted from Stansberry et al. (2024) and Reis et al. (in review).

Item	Gestation		Lactation	
	Control	Moringa	Control	Moringa
**Ingredients, %**				
**Corn**	79.6	75.2	58.8	54.2
**Soybean meal, 47% CP**	15.3	14.5	34.0	33.1
**Moringa dried leaf powder**	0.00	4.00	0.00	4.00
**Swine grease**	1.00	2.38	3.00	4.70
**Limestone**	1.31	1.12	1.43	1.23
**Monocalcium phosphate**	1.06	1.08	1.34	1.37
**Salt**	0.50	0.50	0.50	0.50
**Phytase^b^**	0.10	0.10	0.10	0.10
**Vitamin + TM premix^c^**	0.15	0.15	0.15	0.15
**Choline chloride (60%)**	0.10	0.10	0.10	0.10
**CarniChrom^d^**	0.01	0.01	0.01	0.01
**Availa sow^e^**	0.075	0.075	0.075	0.075
**Clarify^f^**	0.33	0.33	0.10	0.10
**Mycotoxin aide^g^**	0.25	0.25	0.25	0.25
**Titanium premix^h^**	0.20	0.20	0.20	0.20
**Total**	100	100	100	100
**Calculated analysis**				
**ME, Kcal/kg**	3293	3293	3359	3360
**Crude protein, %**	13.9	14.1	21.1	21.2
**Total lysine, %**	0.65	0.67	1.15	1.17
**SID^i^ Lys, %**	0.55	0.55	1.00	1.00
**Calcium, %**	0.75	0.75	0.90	0.90
**Total *P*, %**	0.54	0.54	0.68	0.68
**ATTD *P*, %**	0.35	0.35	0.45	0.45
**Analyzed composition, %^i^**				
**Ash**	5.63	5.59	6.82	6.90
**Crude protein**	14.5	14.5	20.5	21.5
**Crude fiber**	2.22	2.33	2.71	3.03
**Crude fat**	3.16	5.39	5.48	7.73
**Lysine**	0.65	0.72	1.17	1.30
**Methionine**	0.22	0.23	0.31	0.32
**Cysteine**	0.22	0.24	0.32	0.33
**Threonine**	0.50	0.53	0.78	0.84
**Tryptophan**	0.20	0.19	0.30	0.32
**Isoleucine**	0.59	0.63	0.94	1.02
**Leucine**	1.37	1.40	1.82	1.89
**Valine**	0.67	0.71	1.02	1.10

a Gestation diets were fed from approximately day 100 of gestation until farrowing and lactation diets were fed from day of farrowing to weaning.

b Phyzyme (Danisco Animal Nutrition, Marlborough, U.K.) providing 600 phytase units (FTU)/kg.

c Provided per kg of diet: vitamin A, 9,010 IU; vitamin D3, 2,254 IU; vitamin E, 60 IU; vitamin K, 2.21 mg; riboflavin, 7.1 mg; pantothenic acid, 20.1 mg; niacin, 39.9 mg; thiamine, 2.21 mg; and B12, 0.037 mg; biotin, 0.24 mg; folic acid, 1.74 mg; pyridoxine, 4.00 mg; iron, 100.0 mg; zinc, 120.0 mg; manganese, 50.0 mg; copper, 20.0 mg; and iodine, 0.70 mg; selenium, 0.3 mg.

d Rovimix CarniChrom (DSM—Firmenich Animal Nutrition, Union MO), provided per kg of diet: chromium, 0.20 mg; and carnitine 49.6 mg.

e Availa Sow (Zinpro Corporation, Eden Prairie, MN) is an organic trace mineral amino acid complex that provides 50.0 ppm zinc, 20.0 ppm manganese, 10 ppm copper.

f Clarifly larvicide (Central Life Sciences, Schaumburg, IL) provided 22.3 and 6.75 ppm diflubenzuron in the gestation and lactation diets, respectively.

g Mycotoxin aide: Defusion Plus, Cargill Animal Nutrition, Minneapolis, MN, USA.

h Titanium premix contained 0.1% titanium dioxide and 0.1% fine ground corn.

i Diet analysis was performed at University of Missouri Experiment Station Chem Lab.

The ECP system was programmed to flush 2.0 L of chilled water over 30 seconds when any of the three temperature sensors reached 26°C. Further details on ECP function and design are available in prior publications (Cabezon et al. 2017; Cabezón et al. 2017; Schinckel and Stwalley 2019). To simulate moderate heat stress, barn temperatures gradually increased to mimic a hot summer day (Fig. 1), starting on gestation day 110. The temperature increased hourly from 0800, reaching 32°C by 1100, and remained there until 1700. Afterward, the temperature gradually decreased, reaching 26°C by 2000 and maintained overnight until 0800. Overhead fluorescent lights in the farrowing barn were on from 0600 to 2200, and heat lamps with 250-Watt red bulbs were used from 2200 to 0600 to maintain piglet temperature (Savant Technologies LLC, Ohio; Producer’s Pride, TN; and Feit Electric CA, USA). Two sows were removed from the study due to illness, hypophagia, and poor milk production post-farrowing. One sow, initially assigned to the *Moringa* diet, refused to eat and was switched to the control diet, after which she performed normally. Consequently, the final distribution of sows across treatments were HS + CS (*n* = 12), HS + M (*n* = 11), ECP + CS (*n* = 13), and ECP + M (*n* = 10).

### Milk sampling and collection: colostrum, transitional, and mature milk stages

Colostrum was manually collected from sows within two hours following the birth of the first neonate, on lactation day 0 (D0), during active farrowing when oxytocin levels are naturally elevated. In addition to colostrum, milk samples were also collected on lactation days 3 (D3) and 14 (D14) to represent transitional and mature milk stages. For colostrum collection, at least 25 mL was manually expressed from all mammary glands into a 50 mL conical tube, with gentle massage applied to stimulate milk flow. On lactation days 3 and 14, at least 30 mL of milk was collected into a 50 mL conical tube. Prior to milking on days 3 and 14, 0.5 mL of oxytocin (VetOne; Boise, ID; 20 USP.mL-1) was injected into the ear vein using a tuberculin syringe and needle. Milk collection occurred within one minute of oxytocin administration, and teats were massaged to encourage milk flow. Milk was collected from multiple teats to ensure a homogeneous sample, and piglets remained with the sow during the collection. After collection, 1 mL aliquots were placed into 13 microcentrifuge tubes, and 5 mL was transferred to a 15 mL conical tube for further analysis. The samples were initially stored at -20°C before being transferred to -80°C for long-term storage.

### Metabolomic analysis

Multiple reaction monitoring profiling used to identify the different metabolites (de Lima et al. 2018; Dipali et al. 2019; Suarez-Trujillo et al. 2020) in sow milk was performed in the Metabolite Profiling Facility at Bindley Bioscience Center at Purdue University. Using the Bligh and Dyer method, metabolites were extracted from 50 µL of sow colostrum (*n* = 44) or milk (*n* = 43) across D3 and D14 (Bligh and Dyer 1959). The polar phase of extracts was vacuum-dried and then 350 µL of acetonitrile/deionized water (50:50) was used to resuspend the samples, then resuspended samples were diluted 50X in the injection solvent consisted of acetonitrile, methanol, and ammonium acetate in a ratio of 3:6.65:0.35 (v/v/v) for metabolite analysis. Samples were injected using the micro-autosampler (G1377A) of the QQQ6410 triple quadrupole mass spectrometer with an ESI ion source (Agilent Technologies, San Jose, CA). Acetonitrile containing 1% formic acid at 10 μL/min was used between injections (CapPump G1376A, Agilent Technologies, San Jose, CA). Each sample was profiled for the presence of 367 MRM in negative mode and 317 MRM in positive mode (Ferreira et al. 2016; Cordeiro et al. 2017; Yannell et al. 2018; Xie et al. 2019), based on the previously developed method (Edwards et al. 2021). At the completion of this analysis, 326 MRM profiles selected for downstream analysis were found to be 1.3-fold greater than the blank in at least one sample (Tables S1 and S2; doi : 10.4231/2RPX-2D11; Beckett et al. 2025). The data were standardized by sum of intensities within each ion mode (positive and negative; Table S3 doi : 10.4231/2RPX-2D11; Beckett et al. 2025).

### Data and statistical analysis

Data visualization and statistical analysis were conducted using MetaboAnalyst 6.0 (Pang et al. 2024). After uploading the data, auto-scaling normalization was applied. The overall effect of lactation day, diet, ECP and parity on metabolite profiles, were analyzed using one-way ANOVA with false discovery rate (FDR) or raw *p*-value < 0.05. ANOVA analysis was followed by Fisher’s LSD post-hoc test. T-test analysis with multiple corrections in MetaboAnalyst 6.0 was used to evaluate the effects of diet, ECP, and parity within a lactation day, as well as differences between lactation days. Principal component analysis (PCA) and hierarchical cluster (heat map and dendrogram) analysis were used for visualization of metabolite data across time. To distinguish increased/decreased metabolites between days of lactation, Volcano plot in MetaboAnalyst 6.0 was used, which combines results from Fold Change (FC) Analysis and T-tests into one single graph, allowing to intuitively select significant features based on either biological significance, statistical significance, or both. The total abundance of metabolites is presented as fold changes in relation to the sum of the normalized ion intensities of each sample. Significance was defined at *P* ≤0.05 and tendencies identified at 0.05 < *P* ≤ 0.10.

Pathway analysis was conducted using MetaboAnalyst 6.0, which integrates pathway enrichment and topology analysis, allowing for the identification of key metabolic pathways associated with the observed metabolite changes. Metabolite data were uploaded to the pathway analysis module, where compounds were mapped to pathways using the Kyoto Encyclopedia of Genes and Genomes (KEGG) database. The analysis incorporated enrichment statistics to determine the significance of pathway alterations and topological measures to assess the impact of each metabolite within a given pathway. Both *p*-value and impact were used to select pathways for discussion to ensure a comprehensive analysis by identifying statistically significant changes (*p*-value) while prioritizing pathways with the greatest biological relevance (impact), as pathways with higher impact values are those where key metabolites play a central role, reflecting more substantial metabolic disturbances. The specific pathway analysis parameters chosen were scatter plot, hypergeometric test, relative-betweenness centrality, and used all compounds in the selected pathway library. The selected pathway library was *Sus scrofa* (pig; KEGG). All supplementary files can be found at the Purdue University Research Repository using DOI: doi : 10.4231/2RPX-2D11.

## Results

### Changes in metabolites between D0, D3 and D14 samples

**Fig. 2.**
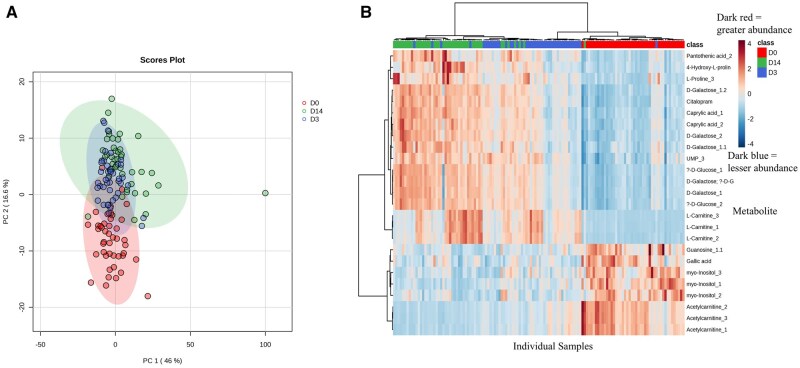
Principal component analysis of milk metabolites across lactation days (D0, D3, and D14). a) principal component analysis (PCA), and b) hierarchical cluster analysis represented by a heatmap with a dendrogram of metabolites detected in colostrum (D0), transitional milk (D3), and mature milk (D14). Sow colostrum (*n* = 44) and milk for D3 and D14 (*n* = 43 for both days) metabolites were analyzed with multiple reaction monitoring profiling. 2D scores plot of PCA was generated based on individual sample abundance of 326 detected metabolites. Hierarchical clustering demonstrates the top 25 most abundant metabolites out of the 279 differentially abundant metabolites.

Scores plots of principal component analysis (PCA, Fig. 2a) and the heat map with associated dendrogram of hierarchical cluster analysis (Fig. 2b) indicated that day of lactation had a significant effect on milk metabolites. In particular, D0 samples in the PCA scores plots showed distinct clusters from the other 2 days. Transitional milk (D3) samples were intermediate to D0 and mature milk (D14), with a seemingly greater overlap between D3 and D14. Similarly, hierarchical cluster analysis of the top 25 differential metabolites demonstrated that D14 separated from D0 and D3, and then D0 from D3. One-way ANOVA determined that 279 lipids were statistically different (FDR < 0.05) between days.

Subsequent T-test (FDR < 0.05) analysis identified 148 metabolites that significantly differed between D0 and D3, with 55 metabolites higher in abundance and 93 lower in D3 compared to D0 samples (Table 2). Between D3 and D14, 169 metabolites were significantly altered (FDR < 0.05), with 148 higher in abundance and 21 lower in D14 compared to D3 (Table 3). The comparison between D0 and D14 revealed 265 metabolites significantly altered (FDR < 0.05), with 180 more abundant and 85 less in D14 (Table 4).

**Table 2.** Metabolites significantly altered across lactation day 3 (D3) compared to day 0 (D0). Metabolites significantly different in abundance by false discovery rate (FDR < 0.05) between lactation days D3 and D0 based on *T*-test analysis. Values are mean Log2 fold-change (FC) with the direction of comparison D3/D0. Color-code shading indicates relative abundance of metabolites between the days of lactation, with the most abundant shaded deepest red and least abundant deepest blue.

Increased and decreased metabolites in D3 compared to D0		
Metabolite	log2(FC)	FDR
**L-Carnitine**	2.04	7E-18
**L-Proline**	1.02	6E-08
**Cytidine**	0.80	2E-06
**D-Galactose; D-Glucose**	0.73	6E-16
**D-Glucose**	0.71	6E-16
**L-Glutamate; Glutamic acid**	0.67	5E-11
**Citalopram**	0.64	2E-15
**L-Lysine**	0.63	7E-09
**D-Galactose**	0.57	4E-16
**4-Hydroxy-L-proline**	0.46	3E-09
**Caprylic acid**	0.45	2E-11
**Maltose**	0.42	3E-07
**Taurine**	0.42	5E-08
**Hypoxanthine**	0.41	3E-06
**D-Lactose**	0.41	5E-09
**L-Lysine; Glutamine**	0.39	1E-04
**Pantothenic acid**	0.36	9E-09
**Cytosine**	0.32	2E-06
**L-Isoleucine; L-Leucine**	0.31	1E-03
**Maltose; Sucrose**	0.28	6E-05
**ADP-Rib**	0.26	3E-02
**L-Phenylalanine**	0.25	2E-05
**Methionine**	0.25	1E-03
**Valine**	0.25	2E-02
**L-Isoleucine**	0.24	2E-03
**DL-Homoserine**	0.23	1E-02
**D-Fructose; D-Glucose**	0.16	5E-02
**Glutamine**	0.10	2E-05
**Bilirubin**	0.08	2E-06
**Sucrose**	0.07	2E-07
**Maltose**	0.07	3E-07
**5'-CMP**	0.07	2E-04
**UMP**	0.03	9E-11
**L-Tyrosine**	−0.07	6E-08
**Thymine**	−0.11	1E-02
**L-Tryptophan**	−0.12	5E-03
**Dihydroxyacetone phosphate; Glyceraldehyde 3-phosphate**	−0.14	1E-02
**L-Phenylalanine**	−0.15	7E-04
**NAD**	−0.15	3E-02
**Malic acid**	−0.16	1E-03
**Shikimic acid; Aconitic acid**	−0.16	4E-03
**Quinic acid**	−0.17	7E-05
**Lactic acid**	−0.18	2E-03
**Inosine**	−0.18	1E-06
**L-Glutamate**	−0.19	2E-06
**Citric acid**	−0.19	3E-02
**Glyceric acid**	−0.19	4E-06
**D-Mannitol; Dulcitol (ie Galactitol); D-Sorbitol**	−0.19	3E-06
**D-Sorbitol**	−0.20	1E-07
**L-Aspartic Acid**	−0.20	7E-04
**2-hydroxy-butanoic acid**	−0.20	3E-05
**Ribulose; Ribose; D-(-)-Arabinose; L-(+)-Arabinose; D-(+)-Xylose**	−0.20	3E-08
**D-Mannitol**	−0.20	4E-07
**D-Ribulose 5-phosphate**	−0.21	2E-07
**Isocitrate**	−0.22	2E-04
**Caffeine**	−0.22	3E-06
**Thymidine**	−0.22	8E-07
**adipic acid**	−0.22	1E-05
**Pyruvate**	−0.22	2E-07
**Oxoglutaric acid**	−0.22	3E-07
**Shikimic acid**	−0.22	4E-07
**Oxaloacetate**	−0.22	7E-05
**Stearic acid**	−0.23	4E-06
**Allantoin**	−0.25	3E-08
**fumaric acid**	−0.25	2E-09
**Globoidnan A**	−0.27	4E-10
**UDP-Glu**	−0.28	6E-04
**Maltose**	−0.28	1E-03
**Arginine**	−0.29	1E-07
**D-Ribulose 5-phosphate; D-Xylulose 5-phosphate**	−0.30	2E-06
**Creatinine**	−0.30	3E-08
**D-Fructose 6-phosphate**	−0.30	4E-05
**UDP**	−0.31	9E-07
**D-Biotin**	−0.31	8E-09
**Uracil**	−0.32	2E-02
**O-Phosphoethanolamine**	−0.33	5E-06
**Raffinose**	−0.34	2E-08
**Aconitic acid**	−0.35	2E-09
**Guanine**	−0.36	5E-02
**myo-Inositol**	−0.43	4E-15
**Creatine**	−0.44	2E-09
**Gallic acid**	−0.49	3E-12
**2-isopropylmalate; 2-Isopropylmalic acid**	−0.51	3E-05
**Guanosine**	−0.55	4E-10
**Uridine**	−0.66	4E-07
**Acetylcarnitine**	−0.1.20	6E-13

**Table 3.** Metabolites significantly altered across lactation day 14 (D14) compared to day 3 (D3). Metabolites significantly different in abundance by false discovery rate (FDR < 0.05) between lactation days D14 and D3 based on *T*-test analysis. Values are mean Log2 fold-change (FC) with the direction of comparison D14/D3. Color-code shading indicates relative abundance of metabolites between the days of lactation, with the most abundant shaded deepest red and least abundant deepest blue.

Increased and decreased metabolites in D14 compared to D3		
Metabolite	log2(FC)	FDR
**L-Lysine**	0.60	2E-03
**L-Glutamate; Glutamic acid**	0.58	0.03
**Cholesterol**	0.58	0.01
**NAD**	0.55	1E-05
**Histidine**	0.54	1E-03
**Stearic acid**	0.54	0.01
**Oxaloacetate**	0.53	0.03
**Anthranilic acid**	0.51	1E-08
**L-Proline**	0.50	5E-04
**L-Aspartic Acid**	0.50	5E-03
**Alanine**	0.49	0.01
**Succinic acid**	0.49	0.02
**Palmitic acid**	0.48	3E-03
**L-Lysine; Glutamine**	0.48	2E-03
**6-Hydroxynicotinic acid**	0.43	0.02
**Margaric acid**	0.42	0.03
**Dulcitol (ie Galactitol)**	0.42	0.02
**Citrulline**	0.42	0.02
**4-Hydroxy-L-proline**	0.41	2E-05
**Ranitidine**	0.41	0.01
**Pyruvate**	0.41	0.03
**D-Lactose**	0.41	3E-03
**Histidine**	0.40	1E-03
**L-Serine**	0.40	0.01
**Ornithine**	0.39	0.01
**Maltose**	0.39	7E-05
**D-Sorbitol**	0.39	0.01
**Glutamine**	0.38	0.01
**3-Indoleacetic Acid**	0.37	0.01
**D-Fructose 6-phosphate**	0.37	0.03
**Lauric acid**	0.37	0.01
**Sphingosine**	0.37	0.01
**Valine**	0.37	0.03
**DL-Homoserine**	0.37	0.01
**Sucrose**	0.37	9E-05
**Shikimic acid**	0.36	0.01
**Citric acid**	0.36	0.02
**Thiamine monophosphat**	0.36	0.01
**D-Galactose**	0.36	1E-08
**Theanine**	0.35	5E-03
**2-Isopropylmalic acid; 2-isopropylmalate**	0.35	0.02
**L-Threonine; DL-Homoserine**	0.35	0.01
**Thymine**	0.35	0.01
**Aconitic acid**	0.35	0.01
**D-Galactose; D-Glucose**	0.35	2E-06
**Spermidine**	0.35	2E-03
**Caprylic acid**	0.35	1E-08
**D-Mannitol**	0.35	0.03
**fumaric acid**	0.34	3E-03
**Atenolol**	0.34	5E-03
**Asparagine**	0.34	0.01
**D-Glucose**	0.34	2E-06
**Homocysteine**	0.34	0.01
**Glycine**	0.34	0.02
**L-Tyrosine**	0.33	0.01
**Citalopram**	0.33	7E-08
**Kynurenine**	0.33	3E-03
**ADP-Rib**	0.33	1E-03
**Diphenylamine**	0.32	0.01
**Hydroxybupropion**	0.32	4E-03
**Cryptoxanthin**	0.32	0.02
**Retinyl palmitate**	0.32	0.01
**L-Carnitine**	0.32	0.03
**L-Cystine**	0.32	0.01
**Guanosine**	0.32	0.02
**2-Aminoadipic acid**	0.31	2E-03
**CDP**	0.31	0.01
**Creatinine**	0.31	0.05
**Glycerol**	0.31	2E-03
**Allantoin**	0.31	0.01
**L-Phenylalanine**	0.31	0.01
**NADH**	0.31	0.01
**Caffeine**	0.31	0.02
**ADP-Glu**	0.31	0.01
**Pantothenic acid**	0.30	0.01
**Nonanoic acid**	0.30	0.01
**Taurine**	0.29	0.01
**cAMP**	0.29	0.01
**O-Phosphoethanolamine**	0.29	0.01
**Methionine**	0.29	0.01
**Zeaxanthin**	0.28	0.01
**Bilirubin**	0.28	0.01
**Adenine**	0.28	0.03
**Ribulose**	0.28	0.01
**L-Arabitol**	0.27	0.01
**L-Tryptophan**	0.27	0.03
**meso-Erythritol**	0.26	0.01
**Uracil**	0.26	0.04
**L-Isoleucine; L-Leucine**	0.25	0.03
**Hexanoylcarnitine**	0.24	0.03
**Xanthine**	0.24	0.03
**Testosterone propionate**	0.21	0.05
**Malic acid**	0.17	0.04
**D(-)-hydroxy butyric acid**	−0.17	2E-03
**L-Leucine**	−0.18	0.04
**Creatine**	−0.19	0.04
**L-Isoleucine**	−0.20	0.02
**myo-Inositol**	−0.27	1E-04
**UMP**	−0.30	2E-06
**UDP**	−0.32	0.02
**UDP-Glu**	−0.36	3E-05
**Hypoxanthine**	−0.43	2E-04
**Inosine**	−0.58	3E-03
**Propionylcarnitine**	−0.81	2E-03
**Acetylcarnitine**	−0.83	1E-03
**L-2-Aminobutyric acid**	−0.89	0.03

**Table 4.** Metabolites significantly altered across lactation day 14 (D14) compared to day 0 (D0). Metabolites significantly different in abundance by false discovery rate (FDR < 0.05) between lactation days D14 and D0 based on *T*-test analysis. Values are mean Log2 fold-change (FC), with the direction of comparison D14/D0. Color-code shading indicates relative abundance of metabolites between the days of lactation, with the most abundant shaded deepest red and least abundant deepest blue.

Increased and decreased metabolites in D14 compared to D0		
Metabolite	log2(FC)	FDR
**L-Carnitine**	2.36	2E-21
**L-Lysine**	1.23	2E-09
**L-Glutamate; Glutamic acid**	1.14	2E-05
**D-Galactose; D-Glucose**	1.07	4E-25
**D-Glucose**	1.05	2E-25
**Citalopram**	0.97	7E-27
**D-Galactose**	0.93	3E-26
**L-Proline**	0.88	5E-13
**L-Lysine; Glutamine**	0.88	2E-08
**4-Hydroxy-L-proline**	0.87	1E-14
**Caprylic acid**	0.79	7E-27
**Oxaloacetate**	0.68	5E-03
**Cytidine**	0.63	2E-07
**Cholesterol**	0.61	5E-03
**DL-Homoserine**	0.60	5E-05
**NAD**	0.59	9E-09
**Anthranilic acid**	0.58	1E-11
**Succinic acid**	0.58	4E-03
**Citrulline**	0.57	2E-03
**L-Isoleucine; L-Leucine**	0.56	1E-05
**ADP-Rib**	0.55	2E-07
**Margaric acid**	0.55	3E-03
**Dulcitol (ie Galactitol)**	0.55	2E-03
**Palmitic acid**	0.53	1E-04
**2-Isopropylmalic acid; 2-isopropylmalate**	0.52	5E-04
**Cytosine**	0.51	2E-04
**Sucrose**	0.51	2E-09
**Histidine**	0.51	2E-05
**Taurine**	0.50	4E-05
**Maltose**	0.48	5E-09
**Ornithine**	0.48	6E-04
**Valine**	0.48	7E-03
**Cytosine; Hypotaurine**	0.48	2E-02
**Spermidine**	0.47	1E-04
**Ranitidine**	0.47	1E-03
**6-Hydroxynicotinic acid**	0.47	5E-03
**L-Phenylalanine**	0.47	7E-06
**Alanine**	0.47	1E-03
**Methionine**	0.47	1E-06
**L-Serine**	0.46	2E-03
**D-Erythrose 4-phosphate**	0.46	4E-02
**Asparagine**	0.46	2E-04
**D-Lactose**	0.45	7E-11
**L-Threonine; DL-Homoserine**	0.45	9E-04
**Adenine**	0.45	7E-04
**Glycine**	0.44	2E-03
**Citric acid**	0.44	3E-03
**3-Indoleacetic Acid**	0.43	7E-04
**L-Isoleucine**	0.43	2E-05
**Pantothenic acid**	0.43	1E-14
**Lauric acid**	0.42	7E-04
**Glycerol**	0.42	1E-05
**2-Aminoadipic acid**	0.42	1E-05
**Sphingosine**	0.42	1E-03
**Theanine**	0.42	6E-04
**Thiamine monophosphate**	0.39	7E-04
**Hypotaurine**	0.39	2E-04
**Atenolol**	0.39	5E-04
**Hydroxybupropion**	0.39	4E-04
**L-Arabitol**	0.38	4E-04
**O-Phosphoethanolamine**	0.38	2E-04
**Cryptoxanthin**	0.38	3E-03
**Maltose; Sucrose**	0.38	4E-07
**Homocysteine**	0.37	2E-03
**L-Cystine**	0.37	1E-03
**Kynurenine_5**	0.37	4E-04
**Diphenylamine**	0.37	2E-03
**ADP-Glu**	0.36	5E-04
**Retinyl palmitate**	0.36	1E-03
**Nonanoic acid**	0.36	1E-03
**cAMP**	0.36	9E-04
**L-Aspartic Acid**	0.35	5E-04
**meso-Erythritol**	0.35	9E-04
**CDP**	0.34	3E-03
**L-Threonine**	0.34	2E-02
**Zeaxanthin**	0.34	1E-03
**NADH**	0.34	2E-03
**Ribulose**	0.34	5E-04
**Glutamine**	0.31	1E-08
**FAD**	0.29	3E-03
**Testosterone propionate**	0.29	4E-03
**Hexanoylcarnitine**	0.28	8E-03
**Stearic acid**	0.28	7E-03
**GDP**	0.28	2E-02
**Thymine**	0.27	6E-04
**FMN**	0.26	2E-02
**Bilirubin**	0.26	7E-07
**(+)-Tocopherol**	0.25	2E-02
**D-Sorbitol**	0.25	4E-05
**Allantoin**	0.23	9E-05
**D-Fructose; D-Glucose**	0.21	1E-02
**Shikimic acid**	0.21	1E-04
**Xanthine**	0.20	8E-03
**D-Fructose; D-Glucose; D-(+)-Mannose**	0.17	3E-02
**Hypoxanthine**	0.16	2E-04
**L-Tryptophan**	0.14	5E-04
**Elaidic acid**	0.14	2E-03
**D-Mannitol**	0.14	2E-04
**Lactic acid**	0.13	7E-04
**Pyruvate**	0.13	3E-06
**L-Tyrosine**	0.12	3E-04
**5'-CMP**	0.11	7E-04
**Caffeine**	0.10	3E-04
**Aconitic acid**	0.05	8E-07
**Thymidine**	0.02	2E-04
**fumaric acid**	0.01	7E-06
**D(-)-hydroxy butyric acid**	−0.01	1E-04
**2-hydroxy-butanoic acid**	−0.12	2E-02
**adipic acid**	−0.13	1E-02
**L-Glutamate**	−0.14	7E-04
**Glyceric acid**	−0.15	7E-04
**Isocitrate**	−0.15	3E-03
**Quinic acid**	−0.16	5E-04
**Oxoglutaric acid**	−0.16	3E-04
**D-Ribulose 5-phosphate**	−0.16	1E-03
**D-Mannitol; Dulcitol (ie Galactitol); D-Sorbitol**	−0.17	5E-05
**Ribulose; Ribose; D-(-)-Arabinose; L-(+)-Arabinose; D-(+)-Xylose**	−0.18	9E-05
**UMP**	−0.18	6E-14
**Creatinine**	−0.24	1E-06
**Globoidnan A**	−0.24	2E-07
**D-Fructose 6-phosphate**	−0.25	3E-03
**D-Biotin**	−0.26	1E-06
**D-Ribulose 5-phosphate; D-Xylulose 5-phosphate**	−0.26	7E-04
**Uracil**	−0.27	9E-03
**Raffinose**	−0.27	1E-05
**Guanosine**	−0.29	2E-10
**Creatine**	−0.43	3E-10
**Inosine**	−0.44	3E-07
**UDP**	−0.45	1E-05
**Gallic acid**	−0.47	1E-10
**2-isopropylmalate; 2-Isopropylmalic acid**	−0.51	4E-06
**Uridine**	−0.61	1E-05
**myo-Inositol**	−0.63	4E-22
**UDP-Glu**	−0.63	1E-08
**L-2-Aminobutyric acid**	−0.65	3E-02
**Propionylcarnitine**	−0.82	7E-08
**Acetylcarnitine**	−2.03	5E-18

**Fig. 3.**
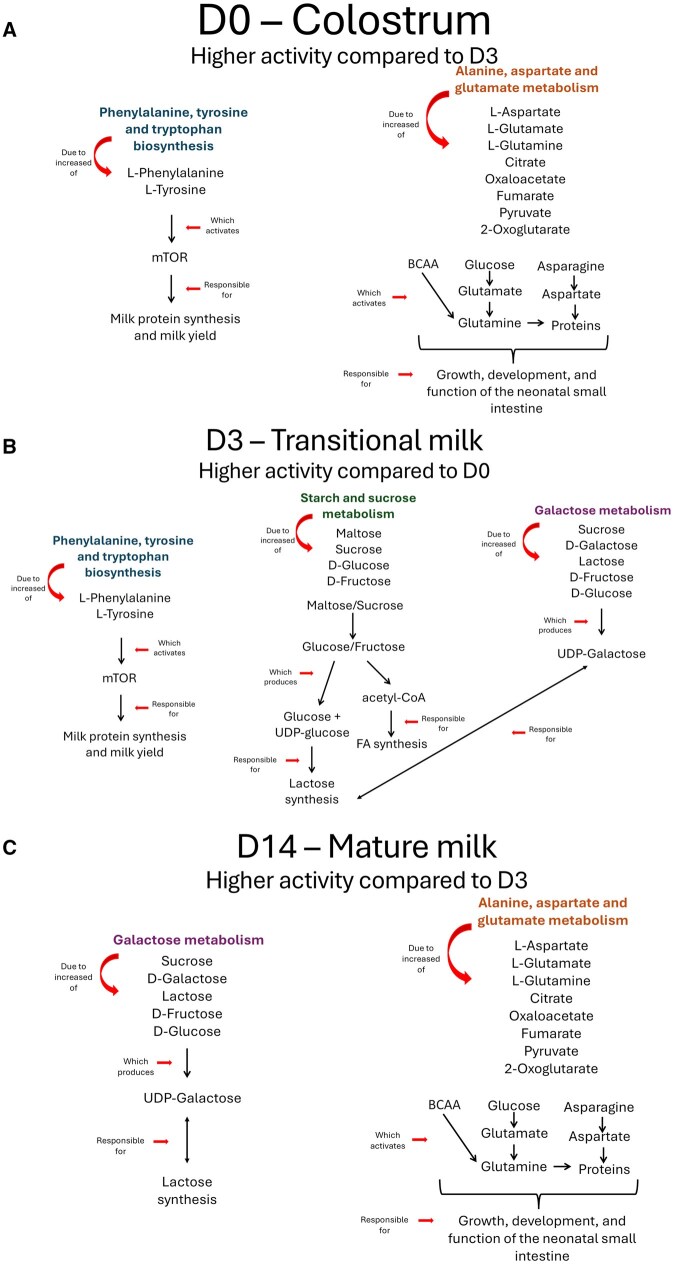
Key metabolic pathways impacted across lactation stages and their role in milk synthesis and neonatal development. The most consistently impacted metabolic pathways across all comparisons, highlighting their role in lactation and neonatal development. Phenylalanine, tyrosine and tryptophan biosynthesis, starch and sucrose metabolism, galactose metabolism, and alanine, aspartate and glutamate metabolism emerged as key pathways influenced by changes in metabolite levels. a) Metabolites increased on D0 compared to D3 were linked to pathways essential for milk protein synthesis and gut development, particularly alanine, aspartate and glutamate metabolism and phenylalanine, tyrosine and tryptophan biosynthesis. b) Metabolites increased on day 3 compared to D0 (D3) and c) day 14 (D14) compared to D3 were primarily associated with enhanced milk protein synthesis, milk yield, lactose and fatty acid synthesis, and neonatal intestinal growth and function. These findings showed the dynamic metabolic adaptations occurring in the mammary gland to support both milk production and neonatal nutritional demands throughout early lactation.

Metabolites significantly greater in D3 milk compared to D0 (FDR < 0.05) enriched pathways (impact > 0.5) including phenylalanine, tyrosine and tryptophan biosynthesis, starch and sucrose metabolism, and galactose metabolism (Table 5, Fig. 3). Metabolites that decreased in milk between D0 and D3 enriched 13 metabolic pathways. Among these pathways (impact > 0.5) were phenylalanine, tyrosine and tryptophan biosynthesis, as well as alanine, aspartate and glutamate metabolism (Table 5, Fig. 3).

**Table 5.** Enriched metabolic pathways and associated metabolites increased or decreased in sow milk on D3 compared to D0. Table presents metabolic pathways significantly enriched (false discovery rate; FDR < 0.05) by metabolites that increased or decreased in milk on day 3 (D3) compared to day 0 (D0). The table includes the pathway name, the corresponding FDR, the impact, which describes the biological relevance of the pathway with higher numbers indicating greater biological significance, and the list of metabolites involved in each pathway. Metabolites detected in our samples appear in red font.

Pathway Name	FDR		Impact	Metabolites
**Pathways enriched with milk metabolites increased in D3 compared to D0**				
**Phenylalanine, tyrosine and tryptophan biosynthesis**	0.03		1.00	Phenylpyruvate; **L-Phenylalanine; L-Tyrosine**; 3-(4-Hydroxyphenyl)pyruvate
**Starch and sucrose metabolism**	0.007		0.55	Cellodextrin; Cellobiose; **D-Fructose; Sucrose**; beta-D-Glucoside; UDP-glucose; D-Glucose 1-phosphate; D-Glucose 6-phosphate; **D-Glucose**; Amylose; alpha, alpha-Trehalose; Maltodextrin; Starch; **Maltose**; Dextrin; Isomaltose; D-Fructose 6-phosphate; alpha-D-Glucose 1,6-bisphosphate
**Galactose metabolism**	0.005		0.54	Stachyose; D-Tagatose 6-phosphate; D-Gal alpha 1->6D-Gal alpha 1->6D-Glucose; **Sucrose**; Raffinose; Melibiose; **D-Galactose**; 3-beta-D-Galactosyl-sn-glycerol; Epimelibiose; Melibiitol; alpha-D-Galactosyl-(1->3)-1D-myo-inositol; alpha-D-Glucose; **Lactose**; D-Glucose 1-phosphate; UDP-alpha-D-galactose; UDP-glucose; alpha-D-Galactose 1-phosphate; alpha-D-Galactose; alpha-D-Glucose 6-phosphate; D-Tagatose 1,6-bisphosphate; **D-Fructose**; **D-Glucose**; Galactitol; Glycerol; D-Mannose; D-Sorbitol; myo-Inositol
**Pathways enriched with milk metabolites decreased in D3 compared to D0**				
**Phenylalanine, tyrosine and tryptophan biosynthesis**	0.04		1.00	Phenylpyruvate; **L-Phenylalanine; L-Tyrosine**; 3-(4-Hydroxyphenyl)pyruvate
**Alanine, aspartate and glutamate metabolism**	0.0001		0.68N-Acetyl-L-aspartate; 2-Oxosuccinamate; **L-Aspartate**; L-Asparagine; D-Aspartate; N-(L-Arginino)succinate; N6-(1,2-Dicarboxyethyl)-AMP; L-Alanine; Succinate semialdehyde; **L-Glutamate**; 4-Aminobutanoate; **L-Glutamine**; Ammonia; 2-Oxoglutaramate; (S)-1-Pyrroline-5-carboxylate; N-Acetylaspartylglutamate; N-Acetylaspartylglutamylglutamate; **Citrate; Oxaloacetate; Fumarate; Pyruvate**; N-Carbamoyl-L-aspartate; Succinate; **2-Oxoglutarate**; Carbamoyl phosphate; D-Glucosamine 6-phosphate; 5-Phosphoribosylamine; beta-Citryl-L-glutamate	

In D14 milk samples, 148 metabolites were more abundant than in D0 samples, enriching 10 pathways. Among these, alanine, aspartate and glutamate metabolism, and galactose metabolism had the highest impact (impact > 0.5) (Table 6; Fig. 3). There were 21 metabolites that were lower in abundance in D14 milk compared to D3, but none significantly enriched any metabolic pathway (FDR > 0.05),

**Table 6.** Enriched metabolic pathways and associated metabolites increased in sow milk on D14 compared to D3. Table presents metabolic pathways significantly enriched (false discovery rate; FDR < 0.05) by metabolites that increased or decreased in milk on day 14 (D14) compared to day 0 (D0). The table includes the pathway name, the corresponding FDR, the impact, which describes the biological relevance of the pathway with higher numbers indicating greater biological significance, and the list of metabolites involved in each pathway. Metabolites detected in our samples appear in red font.

	Pathway Name	FDR	Impact	Metabolites
**Pathways related to the metabolites increased in D14 compared to D3**	Alanine, aspartate and glutamate metabolism	0.0001	0.63	N-Acetyl-L-aspartate; 2-Oxosuccinamate; **L-Aspartate; L-Asparagine**; D-Aspartate; N-(L-Arginino)succinate; N6-(1,2-Dicarboxyethyl)-AMP; L-Alanine; Succinate semialdehyde; **L-Glutamate**; 4-Aminobutanoate; **L-Glutamine**; Ammonia; 2-Oxoglutaramate; (S)-1-Pyrroline-5-carboxylate; N-Acetylaspartylglutamate; N-Acetylaspartylglutamylglutamate; **Citrate; Oxaloacetate**; **Fumarate; Pyruvate**; N-Carbamoyl-L-aspartate; **Succinate**; 2-Oxoglutarate; Carbamoyl phosphate; D-Glucosamine 6-phosphate; 5-Phosphoribosylamine; beta-Citryl-L-glutamate
	Galactose metabolism	0.003	0.54	Stachyose; D-Tagatose 6-phosphate; D-Gal alpha 1->6D-Gal alpha 1->6D-Glucose; **Sucrose**; Raffinose; Melibiose; **D-Galactose**; 3-beta-D-Galactosyl-sn-glycerol; Epimelibiose; Melibiitol; alpha-D-Galactosyl-(1->3)-1D-myo-inositol; alpha-D-Glucose; **Lactose**; D-Glucose 1-phosphate; UDP-alpha-D-galactose; UDP-glucose; alpha-D-Galactose 1-phosphate; alpha-D-Galactose; alpha-D-Glucose 6-phosphate; D-Tagatose 1,6-bisphosphate; D-Fructose; **D-Glucose; Galactitol; Glycerol**; D-Mannose; **D-Sorbitol**; myo-Inositol

The metabolites different in abundance between D0 and D14, greater in D14 milk samples, enriched seven pathways. Among the pathways with the highest impact (impact > 0.5) were alanine, aspartate and glutamate metabolism, and galactose metabolism (Table 7). The 85 metabolites less abundant on D14 compared to D0, significantly (FDR < 0.05) enriched nine pathways, with alanine, aspartate and glutamate metabolism and phenylalanine, tyrosine and tryptophan biosynthesis having the greatest impact scores (impact > 0.5; Table 7, Fig. 3).

**Table 7.** Enriched metabolic pathways and associated metabolites increased or decreased in sow milk on D14 compared to D0. Table presents metabolic pathways significantly enriched (false discovery rate; FDR < 0.05) by metabolites that increased or decreased in milk on day 14 (D14) compared to day 0 (D0). The table includes the pathway name, the corresponding FDR, the impact, which describes the biological relevance of the pathway with higher numbers indicating greater biological significance, and the list of metabolites involved in each pathway. Metabolites detected in our samples appear in red font.

Pathway Name	FDR	Impact	Metabolites
**Pathways enriched with milk metabolites increased in D14 compared to D0**			
**Alanine, aspartate and glutamate metabolism**	0.0001	0.63	N-Acetyl-L-aspartate; 2-Oxosuccinamate; **L-Aspartate; L-Asparagine**; D-Aspartate; N-(L-Arginino)succinate; N6-(1,2-Dicarboxyethyl)-AMP; L-Alanine; Succinate semialdehyde; **L-Glutamate**; 4-Aminobutanoate; **L-Glutamine**; Ammonia; 2-Oxoglutaramate; (S)-1-Pyrroline-5-carboxylate; N-Acetylaspartylglutamate; N-Acetylaspartylglutamylglutamate; **Citrate; Oxaloacetate; Fumarate; Pyruvate**; N-Carbamoyl-L-aspartate; **Succinate**; 2-Oxoglutarate; Carbamoyl phosphate; D-Glucosamine 6-phosphate; 5-Phosphoribosylamine; beta-Citryl-L-glutamate
**Galactose metabolism**	0.0001	0.54	Stachyose; D-Tagatose 6-phosphate; D-Gal alpha 1->6D-Gal alpha 1->6D-Glucose; **Sucrose**; Raffinose; Melibiose; **D-Galactose**; 3-beta-D-Galactosyl-sn-glycerol; Epimelibiose; Melibiitol; alpha-D-Galactosyl-(1->3)-1D-myo-inositol; alpha-D-Glucose; **Lactose**; D-Glucose 1-phosphate; UDP-alpha-D-galactose; UDP-glucose; alpha-D-Galactose 1-phosphate; alpha-D-Galactose; alpha-D-Glucose 6-phosphate; D-Tagatose 1,6-bisphosphate; **D-Fructose; D-Glucose; Galactitol; Glycerol; D-Mannose; D-Sorbitol**; myo-Inositol
**Pathways enriched with milk metabolites decreased in D14 compared to D0**			
**Phenylalanine, tyrosine and tryptophan biosynthesis**	0.04	1.00	Phenylpyruvate; **L-Phenylalanine; L-Tyrosine**; 3-(4-Hydroxyphenyl)pyruvate
**Alanine, aspartate and glutamate metabolism**	0.001	0.58	N-Acetyl-L-aspartate; 2-Oxosuccinamate; **L-Aspartate**; L-Asparagine; D-Aspartate; N-(L-Arginino)succinate; N6-(1,2-Dicarboxyethyl)-AMP; L-Alanine; Succinate semialdehyde; **L-Glutamate**; 4-Aminobutanoate; **L-Glutamine**; Ammonia; 2-Oxoglutaramate; (S)-1-Pyrroline-5-carboxylate; N-Acetylaspartylglutamate; N-Acetylaspartylglutamylglutamate; Citrate; Oxaloacetate; **Fumarate; Pyruvate**; N-Carbamoyl-L-aspartate; Succinate; **2-Oxoglutarate**; Carbamoyl phosphate; D-Glucosamine 6-phosphate; 5-Phosphoribosylamine; beta-Citryl-L-glutamate

Across all comparisons, phenylalanine, tyrosine and tryptophan biosynthesis, starch and sucrose metabolism, galactose metabolism, and alanine, aspartate and glutamate metabolism, emerged as the most consistently impacted pathways. Metabolites that increased from D0 to D3 and D14 were strongly associated with milk protein synthesis, milk yield, lactose and fatty acid synthesis, and growth, development, and function of the neonatal small intestine. Similarly, metabolites that increased on D0 relative to D3—L-phenylalanine, L-tyrosine, L-aspartate, L-glutamate, L-glutamine, citrate, oxaloacetate, fumarate, pyruvate, and 2-oxoglutarate—were linked to pathways with milk protein synthesis and gut development, such as alanine, aspartate and glutamate metabolism and phenylalanine, tyrosine and tryptophan biosynthesis, reflecting early postpartum prioritization of immune and developmental support.

### Impact of diet, ECP and parity within lactation days

Analysis of the overall effect of diet, ECP, and parity on small metabolite content of milk across all days found minimal to no influence of these variables at FDR < 0.05. When the stringency was relaxed to raw *p*-value < 0.05, diet had an overall effect on 4 metabolites, ECP impacted 20, and parity impacted 8 (Table 8).

**Table 8.** Overall impact of lactation day, diet, electronic cooling pad (ECP), and parity on milk metabolites. Data were filtered using the interquartile range with 0% to filter out. Metabolites found with one-way ANOVA analysis, false discovery rate (FDR) or raw *p*-value < 0.05, in MetaboAnalyst 6.0.

	Lactation Day	Diet	ECP	Parity
**FDR < 0.05**	279	0	3	2
**Raw *p*-value < 0.05**	282	4	20	8

Given the significant influence of lactation day on milk metabolites, we explored the effects of diet, ECP, and parity within each lactation day. Metabolite levels were not affected by diet, ECP, nor parity within lactation day at FDR < 0.05, however reducing the stringency to a raw *p*-value of 0.05 to identify metabolites potentially influenced by these factors revealed that diet and parity had a greater influence on milk metabolites on D0, while ECP treatment had a greater influence on D3, and parity on D14 (Table 9).

**Table 9.** Influence of diet, electronic cooling pad (ECP), and parity on milk metabolites on lactation days 0, 3 and 14. Data were filtered using the interquartile range with 0% to filter out. This table describes the number of milk metabolites influenced by diet, ECP, and parity across different lactation days. While no significant effects were observed at FDR of <0.05, a less stringent threshold (raw *p*-value < 0.05) revealed that diet and parity had a more pronounced impact on milk metabolite composition on D0. In contrast, ECP treatment exerted a greater influence on metabolite levels on D3, and parity on D14.

	D0			D3			D14		
	Diet	ECP	Parity	Diet	ECP	Parity	Diet	ECP	Parity
**FDR < 0.05**	0	0	0	0	0	0	0	0	0
**Raw *p*-value < 0.05**	21	10	116	3	104	11	1	12	52

On D0, M supplementation significantly altered 21 metabolites and these enriched pathways related to pentose phosphate as well as fructose and mannose metabolism. The 116 metabolites influenced by D0 and parity were mostly elevated in primiparous sows compared to multiparous sows. These metabolites enriched pathways involved in arginine biosynthesis, alanine, aspartate, and glutamate metabolism, pyrimidine metabolism, arginine and proline metabolism, and purine metabolism. In D14 samples, parity altered 52 metabolites which were related with alanine, aspartate and glutamate metabolism, arginine biosynthesis and pyrimidine metabolism through pathway analysis.

The 104 metabolites reduced by ECP in D3 milk samples were associated with pathways including alanine, aspartate and glutamate metabolism, the citrate cycle (TCA cycle), arginine biosynthesis, pyrimidine metabolism, beta-alanine metabolism, and cysteine and methionine metabolism. At D14, parity altered 52 metabolites which were related to alanine, aspartate and glutamate metabolism, arginine biosynthesis and pyrimidine metabolism through pathway analysis.

## Discussion

Milk metabolites varied across the course of lactation reflecting the state of differentiation and metabolic activity of the gland. Metabolites more abundant in colostrum (D0) samples than in transitional (D3) and mature milk (D14) reflected potential direct passage from circulation through paracellular passages that remain open prior to the onset of secretory activation as well as the differential ion balance (Wellnitz and Bruckmaier 2021) created by this state to include higher levels of acetyl-carnitine, uracil, myoinositol and creatine and creatinine. Transitional milk samples were taken as gland completes secretory activation, which is marked by the onset of lactose and de novo fatty acid synthesis. Metabolites higher in abundance in mature milk samples reflect the climbing energetic demands of growing neonates, and metabolic adjustments made in the mammary gland to the increased biosynthetic capacity for protein, lactose, and lipid components. Changes in metabolites likely also reflected developmental needs of the neonates. Due to the dynamic changes in metabolites across the course of lactation, there was no overall effect of parity, ECP, or M supplementation on the metabolite content of sows exposed to moderate heat stress conditions. However, analysis of variable effects within a lactation day demonstrated moderate effects on metabolite content. The M supplementation primarily affected metabolite levels in colostrum related to the pentose phosphate pathway, which is involved in nucleotide synthesis and NADPH production, and fuels biosynthetic processes including fatty acids. Cooling using ECP impacted transitional milk metabolite content by reducing 104 compounds associated with key biosynthetic and energy-generating pathways in D3 samples. These changes suggest that conductive cooling using ECP during peak heat stress may modulate mammary energy demands or substrate utilization during secretory activation.

Similar to findings of others, high levels of creatine, creatinine, acetyl-carnitine and amino acids like valine, as well as pyroglutamic acid and pyruvate were observed in sow colostrum compared to other metabolites detected (Vötterl et al. 2024). Metabolites found more abundant in colostrum than transitional milk reflect their transfer from the blood into mammary secretory products, included among these were acetyl-carnitine, uridine, guanosine, creatinine, and myo-inositol. Creatine is normally present in human breast milk, but it provides only about 9% of an infant’s estimated diet requirement, responsible for neurological health benefits. The remaining creatine needed for neurological health is supplied through de novo synthesis in the infant’s body or from other dietary sources (Edison et al. 2013; Garwolińska et al. 2018). Creatine is spontaneously and irreversibly broken down to creatinine, meaning that the creatinine found in milk largely reflects creatine metabolism (Brosnan and Brosnan 2007). Creatinine may function in neonates to promote neurological development, and the higher levels in colostrum may reflect evolutionary selection at this stage of milk production as it coincides with rapid early neuronal development (Hulsemann et al. 1987; Edison et al. 2013; Spevacek et al. 2015; Poulsen et al. 2022; Mihatsch et al. 2024).

Metabolites greater in colostrum and transitional milk may reflect the timing and needs of early neonatal gut maturation (Qi et al. 2018). Notably, glutamate serves as a primary energy source for intestinal enterocytes, supporting gut barrier function, enhancing nutrient absorption, promoting mucosal growth, and regulating immune responses in neonates (Cabrera et al. 2013; Zhao et al. 2020; Yao et al. 2023). Along with glutamate, higher levels of glutamine, asparagine and intermediates of citrate cycle in colostrum versus transitional milk may ensure quick, balanced nutrient and energy availability for gut development of neonates immediately after birth (Cabrera et al. 2013; Zhao et al. 2020; Yao et al. 2023). This aligns with previous studies indicating that colostrum ingestion increases gut glutamate and aspartate levels in neonates, facilitating early intestinal development (Cabrera et al. 2013; Zhao et al. 2020; Yao et al. 2023).

Prior to closure of the blood-milk barrier, sodium levels are high in milk, which likely draws myo-inositol into the secretory product. Sodium mediates active transport of myo-inositol into and out of cells, being driven by a sodium gradient (DiNicolantonio and O’Keefe 2022). Following secretory activation, sodium levels drop, and levels of glucose and galactose rise in mammary epithelial cells. Glucose inhibits the sodium dependent SMIT2 myo-inositol transporter, and galactose and glucose reduce myo-inositol uptake and incorporation into phospholipids (DiNicolantonio and O’Keefe 2022). Similar to our findings, studies of human breast milk found myo-inositol levels high during early stages of lactation (Paquette et al. 2023). Positive selection for high levels of sodium in mammary secretions in early lactation may be related to the importance of myo-inositol in the neurologic development of neonates, which occurs rapidly in the early postnatal period (Paquette et al. 2023).

Between D0 and D3, the mammary gland undergoes a metabolic shift resulting in increased volume and nutrient delivery, characterized by elevated levels of sugars such as galactose and lactose—key components for energy production and milk synthesis. In sows, lactose concentration increases progressively from approximately 2.9% in colostrum to 5.11% by day 10 of lactation (S. Zhang et al. 2018). A similar pattern is observed in dairy cows, where colostrum contains about 2.5% lactose and rises to ∼5% in mature milk (Wilms et al. 2019). The initially low lactose concentration in colostrum helps reduce osmotic pressure in the neonatal gut, facilitating the efficient absorption of nutrients and immunoglobulins (Wilms et al. 2019). The shift in metabolites from colostrum to mature milk reflects the evolving role of milk—from initially prioritizing immune protection and gut development to progressively supporting the neonate’s increasing energy and growth demands through enhanced nutrient and lactose supply.

The increase in D-fructose, sucrose, D-glucose, maltose, D-galactose and lactose contents on D3 compared to D0 reflects the enhanced capacity of the mammary gland for lactose synthesis (Lin et al. 2016). Lactose is synthesized from glucose and UDP-galactose via the enzyme lactose synthase. Additionally, glucose generates ATP through glycolysis, supporting energy-intensive processes such as protein and fat synthesis in milk. The overall increase in mono- and disaccharide concentrations reflects enhanced lactose synthesis capacity, consistent with the functional role of the mammary gland at this stage of lactation. This metabolic shift supports the role of secretory activation and suggests that glucose uptake and transporter activity are elevated on D3 compared to D0, to meet the energy demands of the piglet.

Metabolites that significantly increased on D3 compared to D0 included, L-phenylalanine and L-tyrosine, likely reflecting the increased capacity of the mammary gland for milk protein synthesis and milk yield. However, our findings contrast with previous studies, which reported a decrease in tyrosine (Tyr) and phenylalanine (Phe) concentrations from D3 of lactation onward (Yao et al. 2023; Vötterl et al. 2024). As lactation progresses and the gland transitions to mature milk synthesis between D3 and D14, metabolic activity stabilizes, and the mammary gland increases uptake and synthesis of Phe and Tyr to meet the sustained demand for milk protein production (Yao et al. 2023). Phe drives protein synthesis in the mammary gland primarily through activation of the mTOR pathway, which promotes formation of the casein translation initiation complex (Guo et al. 2024). Excess of Phe is converted to Tyr by phenylalanine hydroxylase, ensuring adequate Tyr levels to support protein synthesis and contributing to metabolic regulation and hormonal signaling during lactation (Rae and Ingalls 1984).

In mature milk (D14) compared to transition milk (D3) and colostrum (D0), pathways enriched with metabolites that increased included alanine, aspartate and glutamate metabolism and galactose metabolism. As discussed above, increased enrichment of these pathways reflects roles in maintaining milk composition and supporting continued neonatal development (Hellmuth et al. 2018). Glutamate and aspartate play a crucial role in providing nitrogen for amino acid recycling, which is essential for the sustained production of casein and whey proteins-key components of milk-which support gut development. Glutamate contributes to the mammary gland’s energy metabolism by entering the citrate cycle through α-ketoglutarate, facilitating ATP production to meet the energy demands of ongoing milk synthesis (Li et al. 2009). Citrate cycle intermediates, such as citrate, aconitic acid, isocitrate, α-ketoglutarate, succinyl coA, succinate, fumarate, oxaloacetate, and pyruvate, can leave the cycle via cataplerotic reactions to serve as substrates for biosynthesis of glucose, fatty acids, or non-essential amino acids (Owen et al. 2002). The presence of these metabolites highlights the metabolic adaptation of the mammary gland to sustain milk volume and nutritional quality, ensuring the energy supply required for neonatal growth and development throughout lactation.

Across all days, diet, ECP, and parity had relatively small effects on metabolite content when applying a stringent FDR < 0.05 threshold, indicating that these factors do not broadly reshape milk composition. However, when using a raw *p*-value < 0.05, subtle but potentially biologically relevant differences emerged. *Moringa oleifera* supplementation was associated with increases in metabolites involved in energy metabolism and biosynthetic pathways, such as dihydroxyacetone phosphate/glyceraldehyde 3-phosphate (glycolysis intermediates), creatinine (muscle metabolism), and aconitic acid (linked to the citrate cycle) (Reynolds et al. 1971; Rathod et al. 2020; Kierans and Taylor 2024; Ávila et al. 2025). Use of ECP influenced a larger set of metabolites, including amino acids (L-serine, L-tryptophan, L-phenylalanine, valine), nucleotides (inosine, uridine, xanthine, GDP), and energy-related compounds (lactic acid, NAD), suggesting a broader impact on both protein synthesis and energy metabolism possibly suggesting increased or altered metabolic demands, such as during cellular growth, immune activation, or responses to thermal regulation effects, where both protein synthesis and energy metabolism must be tightly coordinated (Lindqvist et al. 2018, Marchingo and Cantrell 2022; Liu et al. 2025). Parity affected metabolites, with primiparous sows showing higher levels of certain oligosaccharides (raffinose), energy intermediates (propionylcarnitine, pentose phosphate pathway intermediates), and creatinine, which may relate to differences in metabolic adaptation and mammary gland function between first-lactation and multiparous animals (Craig et al. 2019; Nuntapaitoon et al. 2019; Gianluppi et al. 2020; Corrales-Hernández et al. 2025). These findings suggest that while the overall impact of diet, ECP, and parity on milk metabolite composition is modest, each factor may selectively modulate specific metabolic pathways.

Previous work has demonstrated that certain dietary strategies-such as altered energy density, fatty acid supplementation, and inclusion of bioactive plant extracts-can modulate sow milk composition, particularly lipid profiles and immunonutrients (Bai et al. 2017). However, evidence regarding the influence of *Moringa* on milk composition in pigs is still limited. Studies of dairy cattle reported changes in milk nutrients using higher inclusion levels of *Moringa* than those applied in our study, suggesting a possible dose-dependent effect (Zeng et al. 2018), with *Moringa* supplementation levels in our study potentially insufficient to generate detectable alterations in milk metabolites.

Although the findings of changes in small metabolites reported between the days reflect changes in functional activities of the mammary gland, there are several limitations to this study including the exploratory approach of MRM-profiling. Although the scanning of mass transitions used to identify small metabolites is highly sensitive, multiple molecules may have similar attributes and thus limits the specificity and true identity of the molecules. Moreover, the lack of paired analysis with proteome and transcriptome data limits the direct functional validation to confirm the mechanistic role of the metabolites identified in milk composition and neonatal development. Also, the study only evaluates three time points (D0, D3, and D14) during lactation. This limited temporal resolution may miss important intermediate metabolic changes and finer details of the transition from colostrum to mature milk.

## List of abbreviations:

CS =: corn soybean meal control diet
D0 =: day zero of lactation; parturition; colostrum samples
D3 =: day 3 of lactation; transition milk samples
D14 =: day 14 of lactation; mature milk samples
DAG =: diacylglycerols
ECP =: electronic cooling pads
ECP+CS =: electronic cooling pads and control diet
ECP+M =: electronic cooling pads and *Moringa* diet
FC =: fold change
FDR =: false discovery rate
HS =: heat stress
HS+CS =: heat stress and control diet
HS+M =: heat stress and *Moringa* diet
KEGG =: kyoto encyclopedia of genes and genomes
MRM =: multiple reaction monitoring
PCA =: principal component analysis
Phe =: phenylalanine
TAG =: triacylglycerols
Tyr =: tyrosine
